# An In Silico Subject-Variability Study of Upper Airway Morphological Influence on the Airflow Regime in a Tracheobronchial Tree

**DOI:** 10.3390/bioengineering4040090

**Published:** 2017-11-16

**Authors:** Yu Feng, Jianan Zhao, Xiaole Chen, Jiang Lin

**Affiliations:** 1 School of Chemical Engineering, Oklahoma State University, Stillwater, OK 74078, USA; jianan.zhao@okstate.edu; 2 School of Energy and Environment, Southeast University, Nanjing 210018, China; xlc@seu.edu.cn; 3 Zhejiang University of Science and Technology, Hangzhou 310023, China; melinj@163.com

**Keywords:** inter-subject variability, computational fluid-particle dynamics (CFPD), lung airflow regime

## Abstract

Determining the impact of inter-subject variability on airflow pattern and nanoparticle deposition in the human respiratory system is necessary to generate population-representative models, useful for several biomedical engineering applications. Thus, the overall research objective is to quantitatively correlate geometric parameters and coupled transport characteristics of air, vapor, and nanoparticles. Focusing on identifying morphological parameters that significantly influence airflow field and nanoparticle transport, an experimentally validated computational fluid-particle dynamics (CFPD) model was employed to simulate airflow pattern in three human lung-airway configurations. The numerical results will be used to generate guidelines to construct a representative geometry of the human respiratory system.

## 1. Introduction

Configurations and dimensions of human respiratory systems may vary significantly among subjects, thereby influencing airflow as well as aerosol transport and deposition. Thus, it is of interest to assess the degree of inter-subject variability to develop population-representative lung-airway models with beneficial applications in toxicology and drug delivery. Such a subject-variability study can establish relationships between morphological dimensions and airflow characteristics, to guide drug inhalation for individuals with different lung physiological characteristics, as well as drug inhaler design with accurate dose delivery. For example, dry-powder inhalers (DPIs) and pressurized metered-dose inhalers (pMDIs) are the most widely used devices for the treatment of asthma and chronic obstructive pulmonary diseases (COPD). In particular, a pMDI is convenient to use, inexpensive, and allows for multi-dose applications [1]. Moreover, the analysis will generate insight into the physiology of the lung and assist in the development of non-invasive diagnostic tools to treat lung diseases by comparing geometric differences between healthy and diseased configurations. The methodology could also be extended to variability studies between humans and animals. Such inter-species variability investigations are essential to provide more realistic scale-up correlations based on different levels of particle deposition in human vs. animal upper lung airways.

Choi et al. [2] pointed out that there are two factors which significantly affect the airflow between subjects, i.e., the constriction ratio of the glottis concerning the trachea and the curvature and shape of the airways. They employed large eddy simulation (LES) for their turbulence modeling. Farkhadnia et al. [3] investigated the geometric influence on the laminar airflow field and particle transport in G3-G6 triple bifurcations with and without partial blockage due to COPD. Johari et al. [4] compared airflow fields in computed tomography (CT)-scan generated and simplified human airway models and found that over-simplified geometries will induce noticeable differences in numerical simulation results. They also stressed that the roughness of the realistic airway walls might influence the airflow field. Xi et al. [5] investigated particle depositions in different mouth-throat models which were reconstructed via modified morphological parameters of four prototypes. They discovered that the degree of realism of the airway models significantly affected particle deposition from the oral cavity to the glottis, while the effect of the oral airway curvature was minor.

In this study, airflow patterns in three human respiratory systems were investigated using a validated computational fluid-particle dynamics (CFPD) model. Numerical simulations were performed for different steady-state inhalation flow rates (Q_in_ = 37 and 75 L/min). The ultimate goal is to identify morphological parameters that significantly influence the airflow and coupled nanoparticle transport characteristics, and pave the way to construct a representative human upper respiratory system geometry. It is the subject-specific upper lung-airway geometry which generates the “inlet conditions” for the rest of the respiratory tract, and hence determines subsequent airflow fields and nanoparticle deposition. In this paper, we test the hypothesis that despite structural differences in human airway geometries, the basic airflow patterns (i.e., local velocity distribution, glottis jet, and turbulence intensity), as well as nanoparticle depositions, are quite similar.

## 2. Materials and Methods

### 2.1. Human Respiratory System Geometries

**Figure 1:**
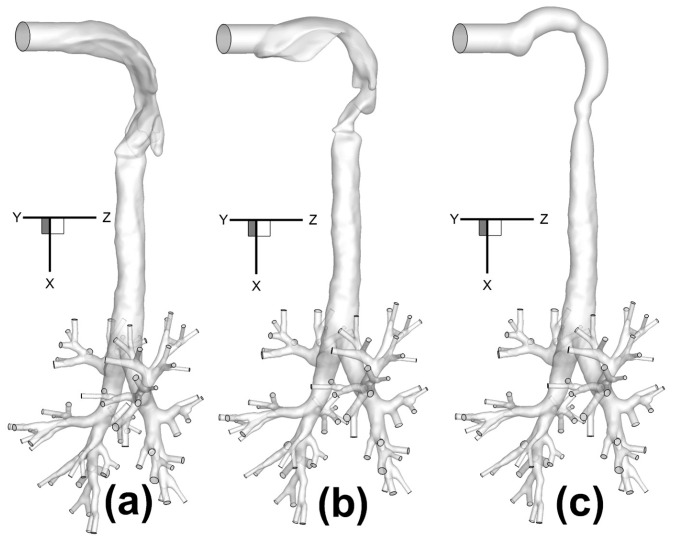
Configurations of human respiratory systems consisting of three human upper airway geometries with the same tracheobronchial tree: (**a**) Geometry A; (**b**) Geometry B; (**c**) Geometry C.

Three human upper airway geometries (Figure 1a–c), were selected and connected to a subject-specific tracheobronchial tree from the middle of the trachea to bifurcations up to Generation 8 (G8) [6]. Each human upper airway geometry contains a 20-mm inhaler inlet, oral cavity, pharynx, larynx, and a portion of the trachea. Inlet centers are all located at (x,y,z) = (0,0,0), and the upper airway geometries end in the plane at x = 0.12 m.

Specifically, Geometry A and Geometry B are two in-house subject-specific human upper airway geometries, which were reconstructed from computed tomography (CT) scans (Figure 1a,b). The tracheobronchial tree used for all three configurations came from the same set of computed tomography (CT) scans of Geometry A. Geometry C, i.e., the idealized human upper airway, is shown in Figure 1c, and was created based on the geometric dimensions measured by Cheng et al. [7].

### 2.2. Upper Airway Morphological Parameters

**Figure 2:**
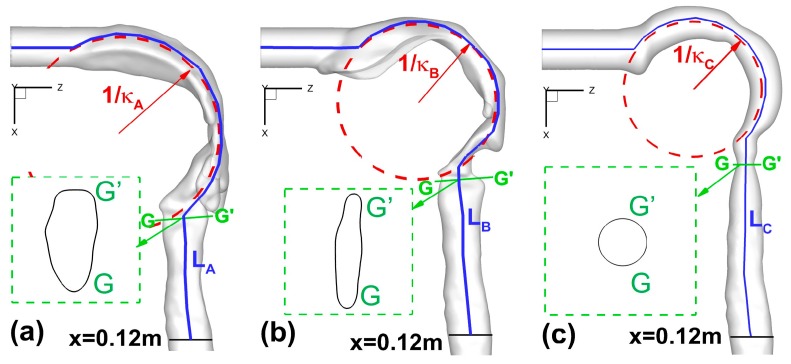
Morphological parameters of the upper airways: (**a**) Geometry A; (**b**) Geometry B; (**c**) Geometry C.

Following the previous publications [2,5,8], four sets of morphological parameters were selected to characterize the inter-subject variability of human upper airways (Figure 2a–c):

1. Total and regional surface areas (*A_tot_* and *A_reg_*) of upper airway geometries;
2. Circularity (*Cr*) and hydraulic diameter (*D_h_*) at different cross-sections;
3. Constriction ratio (*R = A_min_*/*A_in_*) between glottis and mouth inlet openings;
4. Curvature (*κ*) and axial length (*L*) of the upper airway centerlines.

These are critical parameters which may significantly influence airflow pattern and particle deposition as well. Specifically, circularity *Cr* is defined as Equation (1):

(1) $$Cr=\frac{\mathrm{Perimeter}\;\mathrm{of}\;\mathrm{an}\;\mathrm{area}\;\mathrm{equivalent}\;\mathrm{circle}}{\mathrm{Perimeter}\;\mathrm{of}\;a\;\mathrm{luminal}\;\mathrm{area}}$$

The hydraulic diameter *D_h_* is defined as Equation (2):

(2) $$D_{h}=\frac{4\times \mathrm{Luminal}\;\mathrm{area}}{\mathrm{Perimeter}\;\mathrm{of}\;\mathrm{the}\;\mathrm{luminal}\;\mathrm{area}}$$

Table 1 lists geometric parameters at different cross-sections in the three upper airways (see Figure 2a–c). Locations of different cross-sections are also shown in Section 3.2.

### 2.3. Mesh Generation and Independence Test

**Figure 3:**
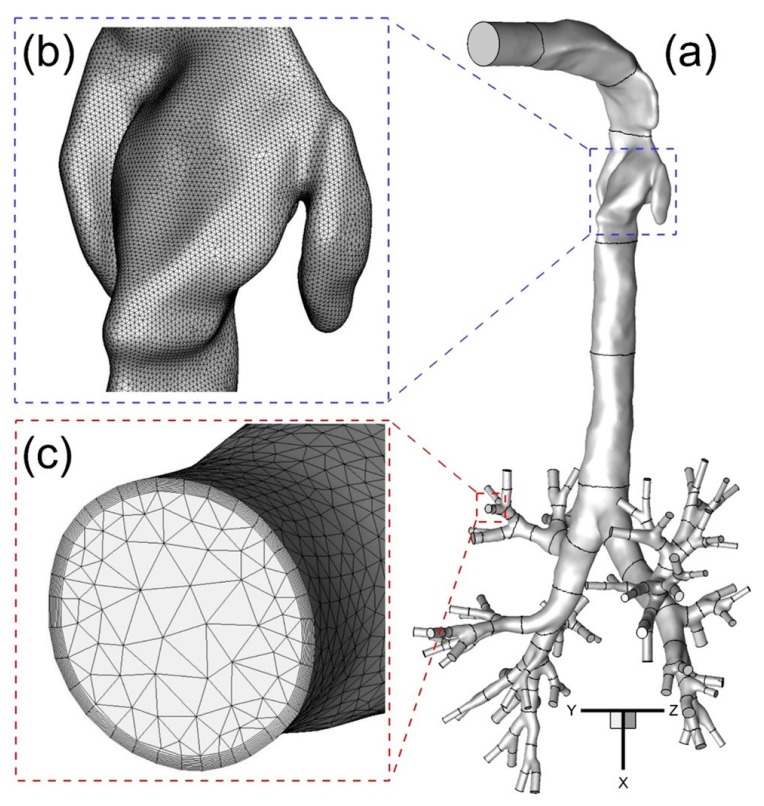
The configuration of subject-specific human upper airway Geometry A from mouth to generation 8 (G8) with tetrahedral mesh including near-wall prism layers: (**a**) Geometry A; (**b**) Surface mesh near the glottis; (**c**) Prism layers at the G6 outlet.

Computational meshes were generated with a multi-layer core region consisting of dense hybrid tetrahedral elements and prism layers. Prism layers were generated near the wall surface to contain the viscous sub-layers fully and to resolve any geometric features present there (Figure 3a–c).

Such high local mesh resolution is also necessary to accurately calculate near-wall derivative values. The thickness of the first prism layer guarantees y^+^ < 1 to capture the laminar and transitional boundary layers correctly, where y^+^ is the dimensionless wall distance (or local near-wall Reynolds number). Specifically, y^+^ < 1 is employed to resolve the viscous sub-layer for optimum accuracy using the shear stress transport (SST) transition model [9,10]. If the y^+^-value is too large, then the transition onset location will not be accurately predicted and will move upstream with increasing y^+^. The mesh topology was determined by refining the mesh until grid independence of the flow field solutions was achieved. As an example, the final mesh of Geometry A contains 11,601,350 elements and 3,403,103 nodes. Mesh details are shown in Figure 3a,b. Considering only the different upper parts of Geometries A to C, the necessary mesh elements are regenerated and tested for the upper airways. The final meshes of B and C contain 10,465,168 and 7,043,696 elements, respectively. The worst skewness is guaranteed at values higher than 0.4.

### 2.4. Governing Equations

The Euler-Lagrange method, enhanced with in-house C programs, is employed to simulate airflow and nanoparticle transport and deposition in the human respiratory systems in future publications. The steady-state airflow in the airways was assumed to be incompressible.

#### 2.4.1 Airflow Field Equations

The airflow dynamics in the respiratory tract is always unsteady and driven by the pressure differences under the action of the cyclic breathing process. In this study, the inhalation conditions are simplified to steady-state. The conservation laws of mass and momentum can be written in tensor form as Equations (3) and (4):

(3) $$\frac{\partial (\rho u_{j})}{\partial x_{j}}=0$$

(4) $$\frac{\partial (\rho u_{i}u_{j})}{\partial x_{j}}=-\frac{\partial p}{\partial x_{i}}+\frac{\partial \tau_{ij}}{\partial x_{j}}+g_{i}$$

where $u_{j}$ represents the fluid velocity, *p* is the pressure, and $g_{j}$ are the body forces, including gravity, electromagnetic force, etc. The viscous stress tensor $\tau_{ij}$ in Equation (4) is given by Equation (5):

(5) $$\tau_{ij}=\mu \left[\left(\frac{\partial u_{i}}{\partial x_{j}}+\frac{\partial u_{j}}{\partial x_{i}}\right)-\frac{2}{3}\delta_{ij}\frac{\partial u_{k}}{\partial x_{k}}\right]$$

With typical inhalation rates, airflow through the oral airway region and the first few generations is incipient turbulent, becoming laminar again at the fourth to sixth generation and remaining so after that. Therefore, a SST transition model was adapted for this study, predicting “laminar-to-turbulent” transition onset, and providing computational efficiency and good accuracy when compared to large eddy simulation. The use of the SST transition model was validated with three-dimensional (3-D) in vitro velocity data in a physical model of a subject-specific human respiratory system [11].

#### 2.4.2. Nanoparticle Dynamics Equations

A Lagrangian frame of reference for the trajectory computations will be employed for nanoparticle dynamics. Assuming nanoparticles to be spheres, in light of the large particle-to-air density ratio and negligible thermophoretic forces, the reduced particle trajectory Equation (6) reads:

(6) $$\frac{d}{dt}(m_{p}u_{i}^{p})==F_{i}^{D}+F_{i}^{L}+F_{i}^{BM}+F_{i}^{G}$$

in which $u_{i}^{P}$ and $m_{p}$ are the velocity and mass of the particle, respectively; $F_{i}^{D}$ represents the drag force; $F_{i}^{G}$ is the gravity [12]; $F_{i}^{BM}$ is the Brownian motion induced force [13,14,15]; and $F_{i}^{L}$ is the Saffman lift force [16].

### 2.5. Numerical Setup

The numerical solution of the governing equations with appropriate boundary conditions was achieved with a user-enhanced, commercial finite-volume based program, i.e., ANSYS Fluent and CFX 18.0 (ANSYS Inc., Canonsburg, PA, USA). The SST transition model was employed to solve the laminar-to-turbulence airflow fields. Numerical simulations were performed on a local Dell Precision T7500 workstation with 40 GB RAM and 12 3.33-GHZ CPUs. The steady-state solution of the flow field was assumed to be converged when the dimensionless mass residual <10^−4^. The user-enhanced C programs, i.e., user-defined functions (UDFs) can perform the following tasks for the full Euler-Lagrange model for future nanoparticle simulations:

(1) Apply anisotropic correction on turbulence fluctuation velocities.
(2) Select two steady-state inlet conditions representing different scenarios of drug inhalation, i.e., 37 L/min and 75 L/min.
(3) Apply uniform gauge pressure at the terminal outlets. It is worth mentioning that the outlet boundary condition is not 100% realistic due to the limitation on measuring pressures at small airways. However, this is the widely used and commonly recognized boundary condition at airway outlets, which also will not influence the subject variability studies of the upper airway morphology influence.

## 3. Results and Discussion

In this section, after validating the CFPD model, results and discussion of the numerical results focus on the following topics:

(1) Mainstream and secondary flow patterns at multiple cross-sections (see Section 3.2. for details), i.e., standard deviation of the velocity.
(2) Length and deviation of laryngeal jet cores from the centerlines.
(3) Local turbulence kinetic energy (TKE) distribution and averaged TKE at cross-sections AA′ to LL′ listed in Table 1.

Morphological parameters such as circularity and constriction ratio that affect airflow patterns are identified as well.

### 3.1. Model Validations

Due to the importance of accurately predicting transitional and turbulent flows in human upper airways, airflow field simulations with the SST transitional model had to be validated. So, the turbulent airflow results were compared with 3-D in vitro data of velocity contours in a subject-specific human respiratory system (i.e., Geometry as shown in Figure 1a), using Magnetic Resonance Velocimetry (MRV) of the identical physical model, i.e., Geometry A [11].

**Figure 4:**
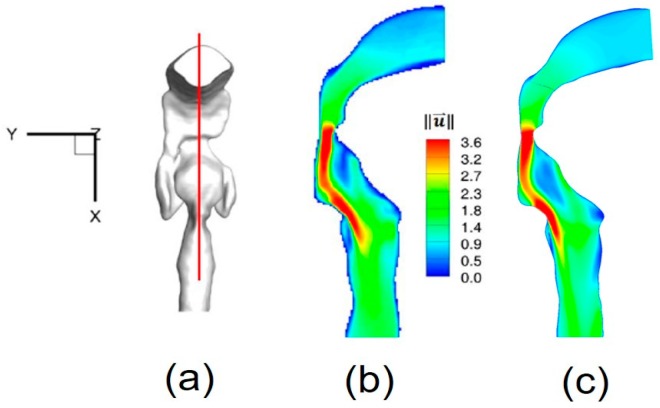
Nondimensionalized velocity magnitude contour comparison at sagittal plane: (**a**) Sagittal plane location; (**b**) Experimental measurement (Reprinted from Reference [11], with permission from Springer); (**c**) Numerical simulation.

**Figure 5:**
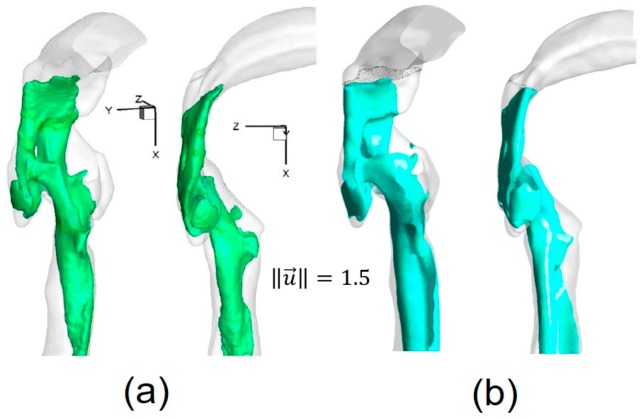
Iso-surfaces of velocity magnitude highlighting the structure of the epiglottal and laryngeal jets: (**a**) Experimental measurement (Reprinted from Reference [11], with permission from Springer); (**b**) Numerical simulation.

The numerical setup for the comparison was the same as for the experimental measurements [11]. For example, the inflow rate was 3.78 L/min of water, and the outlet condition was 20% of the total inflow for each lobe. Contours of non-dimensional local velocity magnitudes $\left\|\vec{u}\right\|$ at the sagittal plane y = 0 (Figure 4a) are shown in Figure 4a–c. Additionally, iso-surfaces, visualizing the epiglottal and laryngeal jet cores ($\|\vec{u}\|=1.5$), are also compared in Figure 5a,b. Good agreements can be observed in both cases.

### 3.2. Secondary Flow Patterns in Cross-Sections and Sagittal Planes

**Figure 6:**
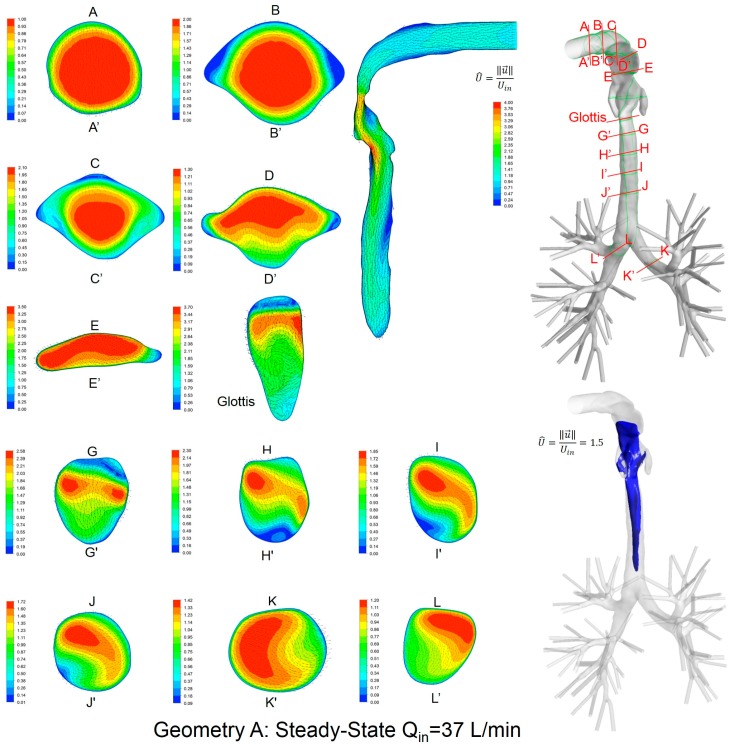
Dimensionless velocity distributions and laryngeal jet core at multiple cross-sections and the sagittal plane of Geometry A under a steady-state inhalation flow rate of Q_in_ = 37 L/min.

**Figure 7:**
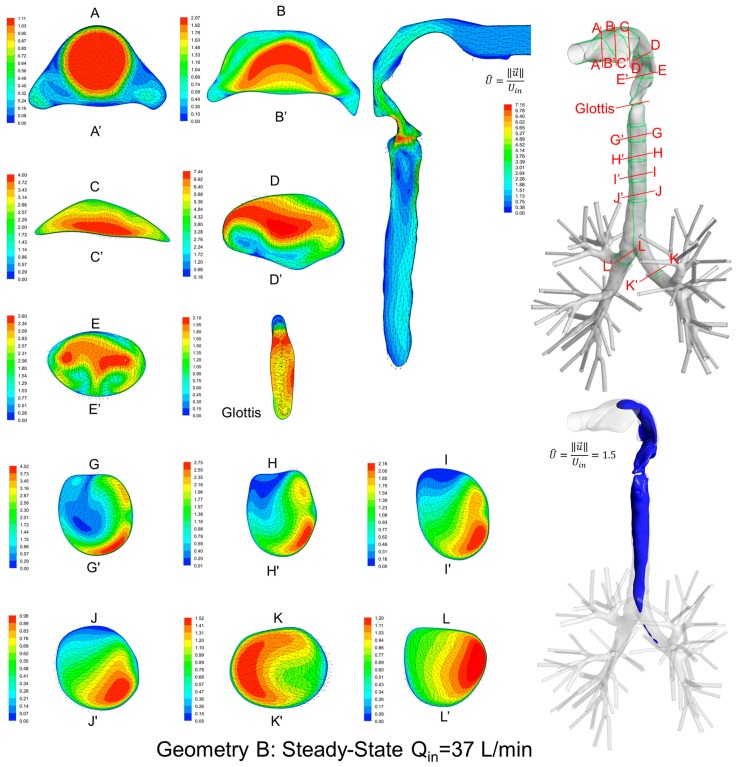
Dimensionless velocity distributions and laryngeal jet core at multiple cross-sections and the sagittal plane of Geometry B under a steady-state inhalation flow rate of Q_in_ = 37 L/min.

**Figure 8:**
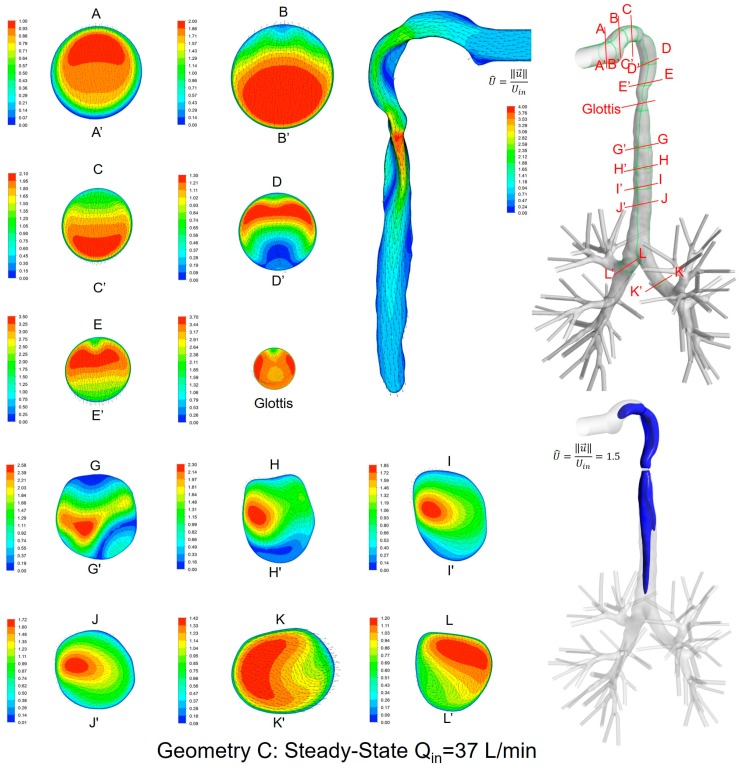
Dimensionless velocity distributions and laryngeal jet core at multiple cross-sections and the sagittal plane of Geometry C under a steady-state inhalation flow rate of Q_in_ = 37 L/min.

Local velocity distributions and secondary flow vectors at different cross-sections and sagittal planes (see Table 1) in the three geometries for Q_in_ = 37 L/min are shown in Figure 6, Figure 7 and Figure 8. As the upper airways end at x = 0.12 m, cross-sections HH′ to LL′ are the same in all three geometries (see Figure 6, Figure 7 and Figure 8).

General flow patterns include:

1. Recirculation regions (i.e., flow separation) exist at the locations with high curvatures and steep lumen changes.
2. A laryngeal jet core is generated in each geometry at the glottis associated with recirculation zones in the trachea.

Distinguished local airflow behavior can be summarized as follows:

1. There are two counter-rotating vortices following the mainstream at both sides in the oral cavities in Geometries A and B, due to the non-circular geometry effect (see AA′ and BB′ in Figure 6, Figure 7 and Figure 8). However, no similar structures exist in the idealized upper airway (Geometry C) with perfect circular outlines at those cross-sections.
2. Due to the large constriction ratio and low circularity at the glottis in Geometry B, high flow resistance around the glottis region breaks up the jet core and spreads it towards the wall (see the contour in the sagittal plane in Figure 7). Recirculation is generated at the upper trachea. However, no such flow patterns are observed in Geometries A and C.
3. Although the lung airway trees employed in the three geometries are the same, mass flow distributions to the left and right lobes are different because of geometric differences among upper airways. For example, Dean’s flows can be observed at KK′ and LL′. However, the local mass flow rate distributions are different, which will potentially influence the fraction of nanoparticles moving into different lung branches.

In summary, Geometry C is not able to quantitatively maintain the complex secondary flow patterns in subject-specific geometries. Nevertheless, it is still a computationally efficient geometry to qualitatively mimic the geometric characteristics and hence the resulting major flow patterns.

### 3.3. Laryngeal Jet Core Structures

**Figure 9:**
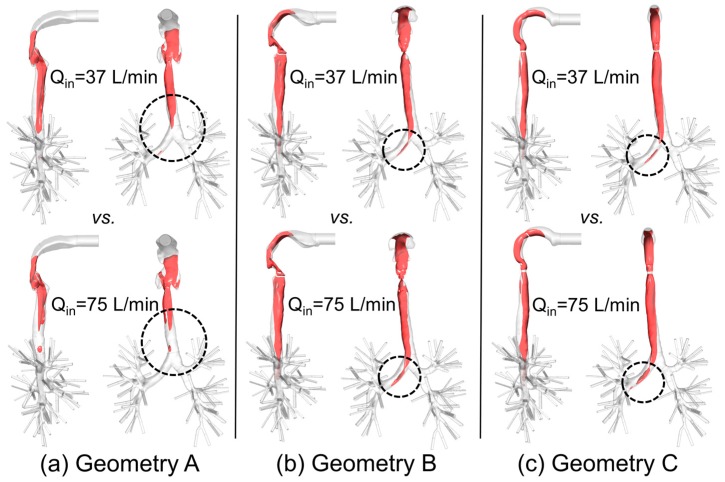
Iso-surfaces of the laryngeal cores $\hat{U}=\|\vec{u}\|/U_{in}=1.5$ in three geometries under two steady-state inhalation flow rates (Q_in_ = 37 L/min vs. Q_in_ = 75 L/min): (**a**) Geometry A; (**b**) Geometry B; (**c**) Geometry C.

To better visualize the laryngeal core structures for $\hat{U}=\|\vec{u}\|/U_{in}=1.5$, Figure 9a–c show the iso-surfaces in three geometries for two steady-state inhalation flow rates, i.e., Q_in_ = 37 L/min and Q_in_ = 75 L/min. Specifically, U_in_ is the average inlet velocity at the mouth.

**Figure 10:**
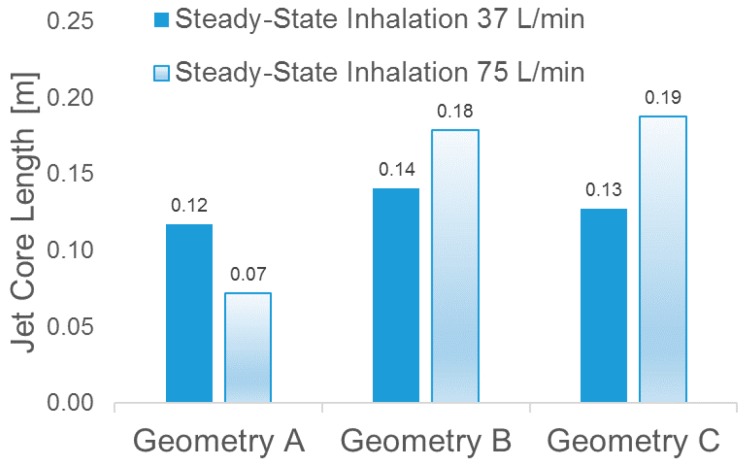
Laryngeal jet core lengths of $\hat{U}=\|\vec{u}\|/U_{in}=1.5$ in the three geometries under two steady-state inhalation flow rates.

In contrast to the conclusion drawn by Choi et al. [2], i.e., “at a higher flow rate, the jet core is found to be shorter and detaches from the tracheal wall”, no decrease in jet-core length was detected between Q_in_ = 37 L/min and Q_in_ = 75 L/min in Geometries B and C (see Figure 10). However, in Geometry A (see Figure 9a), jet core length reduction was found, i.e., higher flow rate induced a shorter length of the laryngeal jet core due to the energy and momentum dissipations when the flow is passing the complex laryngeal region. Specifically, higher inhalation flow rates will lead to stronger impaction, which may or may not induce the redistribution of the jet core region. Additionally, jet-core impaction and breakup depend not only on the inhalation flow rate but also on the jet core orientation with the trachea centerline.

Indeed, Figure 6, Figure 7, Figure 8, Figure 9 and Figure 10 indicate that laryngeal jet cores deviate from the trachea centerlines with highly distinguished patterns in all three geometries for different inhalation flow rates. For example, the jet core impacts the front right trachea in Geometry C, the front left trachea in Geometry B, and the back left trachea in Geometry A, respectively. No common characteristics were discovered, due to the highly different geometric characteristics among the three geometries. Furthermore, Figure 10 indicates that the constriction ratio has an impact on the length of the jet core. Specifically, a small constriction ratio generates a stronger jet core with a longer core length (Geometry C vs. Geometry A).

Because nanoparticles follow the airflow streams in human respiratory systems, it is reasonable to expect that the impingement location and the length of the laryngeal jet core can significantly influence the local deposition of nanoparticles. By guiding patients to operate a drug inhaler with different inhalation flow rates, local deposition of pulmonary nano drugs can be improved for treating diseases in different lung regions.

Another interesting observation is that, despite the axisymmetric shape of the idealized upper airway of Geometry C, the laryngeal cores shown in Figure 8 and Figure 9c are not axisymmetric. This is due to the influence of the reverse flow in the trachea, which conveys the non-axisymmetric geometric effect of the lower lung airways towards the upstream region.

Since the conclusions are diversified between our studies and existing papers [2], more subjects should be included in the future to identify geometric and operational parameters that significantly influence the laryngeal jet core structure.

### 3.4. Turbulence Kinetic Energy (TKE)

**Figure 11:**
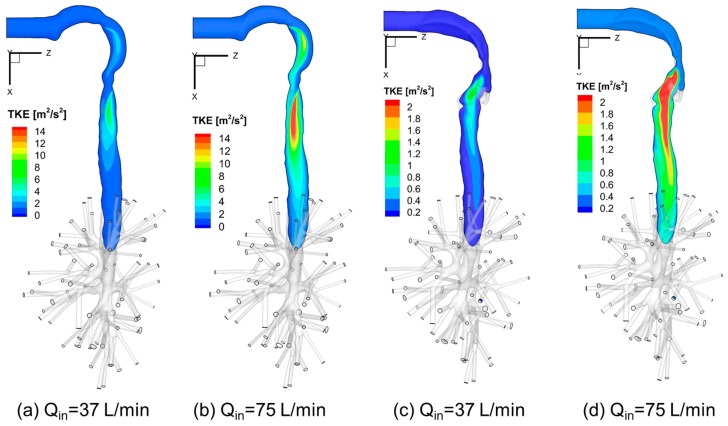
Turbulence Kinetic Energy (TKE) distributions at sagittal planes under different inhalation flow rates: (**a**) Q_in_ = 37 L/min for Geometry C; (**b**) Q_in_ = 75 L/min for Geometry C; (**c**) Q_in_ = 37 L/min for Geometry A; (**d**) Q_in_ = 75 L/min for Geometry A.

Figure 11a–d visualize the local TKE distributions in the sagittal planes of Geometries A and C under two steady-state flow rates, i.e., Q_in_ = 37 L/min and Q_in_ = 75 L/min. Higher TKE locations are where turbulence occurs. Also, a higher flow rate induces larger recirculation flow regions in the trachea and stronger local turbulent fluctuations.

**Figure 12:**
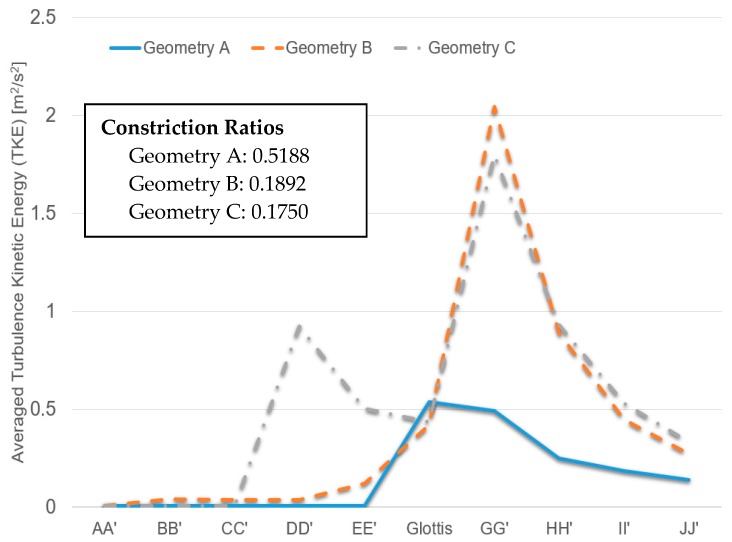
Local turbulence kinetic energies (TKE) under Q_in_ = 37 L/min in the three geometries.

Additionally, Figure 12 shows the averaged TKE of cross-sections AA′ to JJ′ (see Figure 6, Figure 7 and Figure 8 for cross-section locations). The averaged TKE abruptly increases after the constriction of the glottis. It can be observed that the constriction ratio has a significant impact on the higher TKE in the trachea. Indeed, Geometries B and C have similar trends of TKE after the airflows pass the glottis contractions (see Figure 12). In contrast, with a higher constriction ratio, the increase of TKE in Geometry A after the glottis is less steep than that in the other two. The comparison between Geometries B and C also implies that the glottis contraction serves as a transitional flow regulator due to the forced mixing effect when airflow passes the contraction region. Although the averaged TKE at cross-sections differs in the two geometries, their downstream-glottis trends are similar. However, more geometries need to be included in the inter-subject variability study to test this “glottis regulator” concept.

## 4. Conclusions

In this study, numerical inter-subject variability studies were performed for airflows, considering drug inhalation flow rates. It can be concluded that the upper airway morphology has a significant influence on actual flow patterns. This will potentially lead to different deposition patterns of nanoparticles. It was also found that the jet core length does not increase with the increase of inhalation flow rates in all upper airways.

The idealized human upper airway geometry (Geometry C) may not be able to fully represent the naturally asymmetrical flow patterns in subject-specific human respiratory systems. However, Geometry C can be used for initial studies because it qualitatively represents basic flow patterns in human respiratory systems. However, the use of Geometry C may lead to lower local nanoparticle depositions when compared to subject-specific lung-airways.

## 5. Future Work

**Figure 13:**
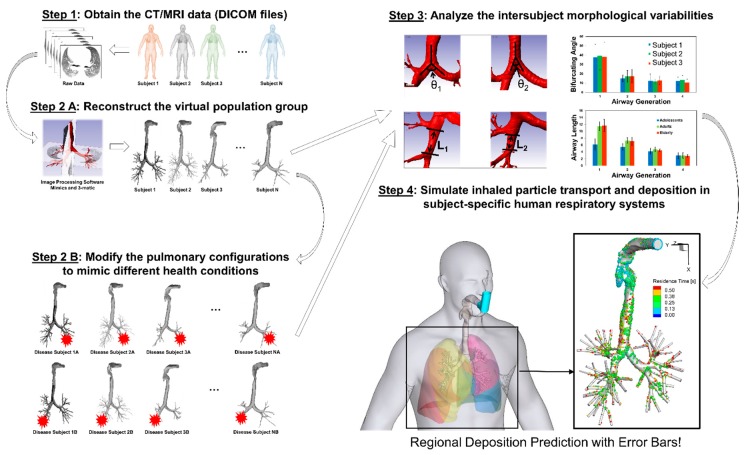
The framework of in silico inter-subject variability study using Computational Fluid-Particle Dynamics (CFPD) models.

Considering the complexity of multiple geometric parameters, it is necessary to include additional airway geometries representing different population groups to generate statistically robust conclusions, i.e., CFPD simulation results with “error bars.” The proposed framework of in silico subject variability study is shown in Figure 13, to pave the way for the development of a virtual human system as the individualized digital twin for personalized pulmonary disease treatment planning.

## Figures and Tables

**Table 1:** Geometric parameters of cross-sections in the three geometries.

**Name**	**Location (m)**			**Area (m^2^)**		
				**A**	**B**	**C**
**AA′**	Z = 0.04			3.277 × 10^−4^	5.846 × 10^−4^	3.491 × 10^−4^
**BB′**	Z = 0.06			4.281 × 10^−4^	6.615 × 10^−4^	2.987 × 10^−4^
**CC′**	Z = 0.08			5.439 × 10^−4^	2.155 × 10^−4^	1.966 × 10^−4^
**DD′**	X = 0.02			4.549 × 10^−4^	2.578 × 10^−4^	1.965 × 10^−4^
**EE′**	X = 0.04			1.619 × 10^−4^	2.068 × 10^−4^	1.033 × 10^−4^
**Glottis**	A	B	C	1.629 × 10^−4^	5.94 × 10^−5^	5.494 × 10^−5^
	X = 0.081	X = 0.064	X = 0.056			
**GG′**	X = 0.1			2.181 × 10^−4^	2.691 × 10^−4^	2.367 × 10^−4^
**HH′**	X = 0.12			2.688 × 10^−4^	2.956 × 10^−4^	2.691 × 10^−4^
**II′**	X = 0.14			2.954 × 10^−4^	3.042 × 10^−4^	2.955 × 10^−4^
**JJ′**	X = 0.16			3.041 × 10^−4^	5.846 × 10^−4^	3.042 × 10^−4^
**Name**	**Perimeter (m)**			**Circularity**		
	**A**	**B**	**C**	**A**	**B**	**C**
**AA′**	0.06469	0.1001	0.0662	0.991908007	0.8562	1.0000
**BB′**	0.07747	0.1189	0.0613	0.946670446	0.7670	0.9999
**CC′**	0.08970	0.0777	0.0497	0.921601854	0.6701	0.9998
**DD′**	0.08835	0.0609	0.0497	0.855688427	0.9349	0.9999
**EE′**	0.06181	0.0528	0.0413	0.7297683	0.9663	0.8722
**Glottis**	0.05291	0.0392	0.0263	0.855112911	0.6955	0.9992
**GG′**	0.05785	0.0583	0.0549	0.904745095	0.9907	0.9943
**HH′**	0.05963	0.0597	0.0597	0.974517927	0.9744	0.9744
**II′**	0.06237	0.0624	0.0624	0.976855682	0.9768	0.9769
**JJ′**	0.06210	0.0621	0.0621	0.995369053	0.9954	0.9954

## References

[1] Kleinstreuer C., Feng Y., Childress E. (2014). Drug-targeting methodologies with applications: A review. World J. Clin. Cases.

[2] Choi J., Tawhai M.H., Hoffman E.A., Lin C.L. (2009). On intra-and intersubject variabilities of airflow in the human lungs. Phys. Fluids.

[3] Farkhadnia F., Gorji T.B., Gorji-Bandpy M. (2016). Airflow, transport and regional deposition of aerosol particles during chronic bronchitis of human central airways. Australas. Phys. Eng. Sci. Med..

[4] Johari N.H., Osman K., Helmi N.H.N., Abdul Kadir M.A.R. (2015). Comparative analysis of realistic CT-scan and simplified human airway models in airflow simulation. Comput. Methods Biomech. Biomed. Eng..

[5] Xi J., Yuan J.E., Yang M., Si X., Zhou Y., Cheng Y.-S. (2016). Parametric study on mouth–throat geometrical factors on deposition of orally inhaled aerosols. J. Aerosol Sci..

[6] Zhang Z., Kleinstreuer C., Feng Y. (2012). Vapor deposition during cigarette smoke inhalation in a subject-specific human airway model. J. Aerosol Sci..

[7] Cheng Y.S., Zhou Y., Chen B.T. (1999). Particle deposition in a cast of human oral airways. Aerosol Sci. Technol..

[8] Choi S., Hoffman E.A., Wenzel S.E., Castro M., Fain S.B., Jarjour N.N., Lin C.L. (2015). Quantitative assessment of multiscale structural and functional alterations in asthmatic populations. J. Appl. Physiol..

[9] Menter F.R. (1994). Two-equation eddy-viscosity turbulence models for engineering applications. AIAA J..

[10] Zhang Z., Kleinstreuer C. (2011). Laminar-to-turbulent fluid–nanoparticle dynamics simulations: Model comparisons and nanoparticle-deposition applications. Int. J. Numer. Methods Biomed. Eng..

[11] Banko A.J., Coletti F., Schiavazzi D., Elkins C.J., Eaton J.K. (2015). Three-dimensional inspiratory flow in the upper and central human airways. Exp. Fluids.

[12] Kleinstreuer C. (2003). Two-Phase Flow: Theory and Applications.

[13] Li A., Ahmadi G. (1993). Deposition of aerosols on surfaces in a turbulent channel flow. Int. J. Eng. Sci..

[14] Feng Y., Kleinstreuer C., Nicolas C., Rostami A. (2016). Computational transport, phase change and deposition analysis of inhaled multicomponent droplet–vapor mixtures in an idealized human upper lung model. J. Aerosol Sci..

[15] Mansour H.M., Rhee Y.-S., Wu X. (2009). Nanomedicine in pulmonary delivery. Int. J. Nanomed..

[16] Saffman P.G.T. (1965). The lift on a small sphere in a slow shear flow. J. Fluid Mech..

